# 2614. Trends in characteristics of influenza-associated hospitalizations among pregnant women, FluSurv-NET 2015-2016 through 2022-2023 seasons

**DOI:** 10.1093/ofid/ofad500.2227

**Published:** 2023-11-27

**Authors:** Catherine Bozio, Dawud Ujamaa, Fatimah S Dawood, Shua Chai, Pam Daily Kirley, Nisha B Alden, Elizabeth Austin, Julia Desiato, Kimberly Yousey-Hindes, Emily Fawcett, Kyle P Openo, Maya Monroe, Patricia A Ryan, Anna Falkowski, Sue Kim, Ruth Lynfield, Melissa McMahon, Kathy Angeles, Sarah Khanlian, Kerianne Engesser, Nancy L Spina, Christina B Felsen, Maria Gaitan, Nancy E Moran, Eli Shiltz, Andie Hendrick, Melissa Sutton, William Schaffner, H Keipp Talbot, Mary Hill, Andrea Price, Alissa O’Halloran, Shikha Garg

**Affiliations:** CDC, Atlanta, Georgia; Centers for Disease Control and Prevention, Atlanta, Georgia; Centers for Disease Control and Prevention, Atlanta, Georgia; California Department of Public Health, Oakland, CA; California Emerging Infections Program, Oakland, California; Colorado Department of Public Health and Environment, Denver, Colorado; Colorado Department of Public Health and Environment - Communicable Disease Branch, Denver, Colorado; Yale School of Public Health, Milford, Connecticut; Yale School of Public Health, Milford, Connecticut; Georgia Emerging Infections Program and Atlanta VA Medical Center, Decatur, Georgia; Georgia Emerging Infections Program and Atlanta VA Medical Center, Decatur, Georgia; Maryland Department of Health, Baltimore, Maryland; Maryland Department of Health, Baltimore, Maryland; Michigan Department of Health and Human Services, Livonia, Michigan; Michigan Department of Health and Human Services, Livonia, Michigan; Minnesota Department of Health, St. Paul, MN; Minnesota Department of Health, St. Paul, MN; NM Emerging Infections Program/University of New Mexico, Las Cruces, NewMexico; New Mexico Emerging Infections Program, Albuquerque, NewMexico; New York State Department of Health, Albany, New York; New York State Department of Health, Albany, New York; University of Rochester, Rochester, NY; Rochester Emerging Infections Program, University of Rochester Medical Center, Rochester, New York; Ohio Dept of Health, Columbus, Ohio; Ohio Department of Health, Columbus, Ohio; Oregon Health Authority, Portland, Oregon; Oregon Health Authority, Portland, Oregon; Vanderbilt University Medical Center, Nashville, Tennessee; Vanderbilt University Medical Center, Nashville, Tennessee; Salt Lake County Health Department, Salt Lake City, UT; Salt Lake County Health Department, Salt Lake City, UT; CDC, Atlanta, Georgia; Centers for Disease Control and Prevention, Atlanta, Georgia

## Abstract

**Background:**

Surveillance data from the Influenza Hospitalization Surveillance Network (FluSurv-NET) detected an increase over time in the proportion of hospitalized women of child-bearing age with laboratory-confirmed influenza who were pregnant. We sought to describe changes in patient characteristics to explain this observed trend.

**Methods:**

FluSurv-NET conducts population-based surveillance for laboratory-confirmed influenza-associated hospitalizations in 13 states. We limited the analysis to women of child-bearing age. Charts were abstracted for all cases in all seasons except the 2022-23 season when a sample of cases were abstracted. Weighted proportions were reported in the 2022-23 season due to sampling. We excluded data from the 2019-20 season (data collection ongoing) and the 2020-21 season (low influenza hospitalization rates).

**Results:**

The proportions of hospitalized women of child-bearing age with influenza who were pregnant were 25.4%-29.4% during the 2015-19 seasons and increased to 35.2%-37.4% during the 2021-23 seasons. Among hospitalized pregnant women with influenza, the proportions with ≥1 respiratory symptom at admission were high (78.2%-84.8%) across the 2015-19 seasons and decreased to 44.0%-56.5% during the 2021-23 seasons. The proportions who were admitted during their third trimester increased from 54.4%-66.8% across the 2015-19 seasons to 75.1%-80.7% during 2021-23 seasons. Among hospitalized pregnant women with respiratory symptoms, the proportions receiving antiviral treatment decreased from the 2015-19 seasons to the 2021-23 seasons (90.3%-95.1% vs. 79.0%-79.6%, respectively).

**Conclusion:**

In recent seasons, changes in characteristics and treatment of hospitalized pregnant women with influenza suggest that influenza may not be the primary reason for admission for an increasing proportion of pregnant women detected in FluSurv-NET since the start of the COVID-19 pandemic; these findings may in part be driven by changes in influenza testing practices. Receipt of antiviral treatment among symptomatic hospitalized pregnant women also decreased in the past two seasons, highlighting the need to improve antiviral use among this group at increased risk for severe illness from influenza.

**Disclosures:**

**Dawud Ujamaa, MS**, GDIT: Employee **Anna Falkowski, Master of Science**, Michigan Department of Health and Human Services: Grant/Research Support **Sue Kim, MPH**, Michigan Department of Health and Human Services: Grant/Research Support

